# The key role of *CYC2* during meiosis in *Tetrahymena thermophila*

**DOI:** 10.1007/s13238-016-0254-9

**Published:** 2016-03-23

**Authors:** Qianlan Xu, Ruoyu Wang, A. R. Ghanam, Guanxiong Yan, Wei Miao, Xiaoyuan Song

**Affiliations:** 1 Hefei National Laboratory for Physical Sciences at the Microscale, University of Science and Technology of China, Hefei, 230026 China; 2 CAS Center for Excellence in Molecular Cell Science, Shanghai, 200031 China; 3 School of Life Sciences, University of Science and Technology of China, Hefei, 230071 China; 4 Key Laboratory of Aquatic Biodiversity and Conservation, Institute of Hydrobiology, Chinese Academy of Sciences, Wuhan, 430072 China; 5 University of Chinese Academy of Sciences, Beijing, 100049 China; 6 Anatomy and Embryology Department, Suez Canal University, Ismailia, 41522 Egypt

**Keywords:** cyclin, meiosis, RNA-Seq, *Tetrahymena thermophila*, homologous recombination

## Abstract

Meiotic recombination is carried out through a specialized pathway for the formation and repair of DNA double-strand breaks (DSBs) made by the Spo11 protein. The present study shed light on the functional role of cyclin, *CYC2*, in *Tetrahymena thermophila* which has transcriptionally high expression level during meiosis process. Knocking out the *CYC2* gene results in arrest of meiotic conjugation process at 2.5–3.5 h after conjugation initiation, before the meiosis division starts, and in company with the absence of DSBs. To investigate the underlying mechanism of this phenomenon, a complete transcriptome profile was performed between wild-type strain and *CYC2* knock-out strain. Functional analysis of RNA-Seq results identifies related differentially expressed genes (DEGs) including *SPO11* and these DEGs are enriched in DNA repair/mismatch repair (MMR) terms in homologous recombination (HR), which indicates that *CYC2* could play a crucial role in meiosis by regulating *SPO11* and participating in HR.

**Electronic supplementary material:**

The online version of this article (doi:10.1007/s13238-016-0254-9) contains supplementary material, which is available to authorized users.

## Introduction

*Tetrahymena thermophila*, one of typical ciliate protozoans, is a unicellular eukaryotic species characterized by its nuclear dimorphism. As each cell contains two differentially developed and functionally distributed nuclei, a diploid germ line nucleus (micronucleus) and a polyploid somatic nucleus (macronucleus) (Collins, 2012). *Tetrahymena* also has two types of life cycle, vegetative growth cycle and meiotic conjugation cycle. In meiotic conjugation cycle, only the micronucleus takes part as one of the similar biological processes which are evolutionarily conserved with multicellular organisms meiosis, except for the extra complicated procedures of several successive mitotic cycles in conjugation after meiotic segregation (Collins, 2012).

Meiosis is a highly conserved process in sexually reproducing eukaryotes. It is a special mode of mitosis, during which the parental diploid chromatins duplicate once followed by two rounds of precise halving of the genome in succession to generate haploid gametes. During gametes production in most gamogenetic species, homologous recombination (HR) occurs in the prophase of meiosis I before the chromosomes segregation starts. At the same time, the chiasmata were formed between aligned homologous chromosomes as a stable physical connection which serves for maintaining the accuracy of chromosome equal segregation (Petronczki et al., 2003).

The recombination is the most protrusive and important process during meiosis I prophase by virtue of its reshuffling function through merging the two parental alleles, generating more diversified progenies, which is of evolutionary significance (Kauppi et al., 2004). Meiotic recombination has at its heart the formation and subsequent repair of DNA double strand breaks (DSBs) (Keeney, 2001). DSB formation is catalyzed by Spo11, which appears to act via a topoisomerase-like reaction to generate a transient, covalent protein-DNA intermediate (Keeney et al., 1997a). The repair of any given meiotic DSB can result in either reciprocal exchange of the chromosome arms flanking the break (a crossover), or no exchange of flanking arms (a noncrossover or parental configuration).

During the early stage of conjugation, two different mating types of *T. thermophila* cells approach each other till the pair formed. Then a rather stable junction formed followed by a course of shape changing of micronuclei and two successions of chromosome segregations. In addition, crescent stage was found to be the analogous to the bouquet stage in multicellular organisms when extremely elongated micronuclei (crescent) were formed at approximately 3 h after conjugation initiation (Loidl and Mochizuki, 2009). This is of great importance for both homologous recombination in prophase and DNA rearrangement during the growth of new macronuclei (Mochizuki and Gorovsky, 2004). On account of the unique life cycle, nuclear dimorphism as well as convenience of inducing meiosis initiation and observable distinctive characteristics summarized at each stage in meiosis, *T. thermophila* could be used as a great and unique research model of meiosis process like *Saccharomyces cerevisiae*.

Cyclin domain is a helical domain for protein recognition, which exists mostly in cyclin superfamily proteins but also in transcription factor II B and Retinoblastoma with different copies and forms of distribution (Gibson et al., 1994). Cyclin domain equips cyclin proteins with key function of conditioning the enzyme activity of cyclin-dependent kinases, which participate in the molecular regulation of the events of cell cycle and transition into the next phases (Zhang et al., 1999). *T. thermophila* possesses 26 known cyclin homologs which are classified into different cyclin groups of functions. Each of *T. thermophila* cyclin protein exhibits unique profile of mRNA expression (TGD website: http://ciliate.org/) (Miao et al., 2009). Among them, 23 of the cyclin homologs have sharp peaks at different time points of conjugation. *CYC2*, an N-terminal cyclin domain containing protein, was grouped into canonical ally and was identified as cyclin D-like proteins using phylogenetic analysis tools (Stover and Rice, 2011). Also, named as COI5 (conjugation-induced gene 5) (Woehrer et al., 2015). *CYC2* gene has no expression at all during logarithm growth when there are only micronuclei mitosis and macronuclei amitosis, as well as in starvation condition. While its expression begins when the meiosis starts about 2 h after the initiation of conjugation and remains high till the end of meiosis II, suggesting its probable role in meiosis process (Miao et al., 2009).

Moreover, yeast B-type cyclins (CLB5 and CLB6) have been reported to have key roles in the initiation of homologous chromosome recombination and the formation of synaptonemal complex during meiosis prophase (Devault et al., 2008; Henderson et al., 2006).

In the advent of Next Generation Sequencing (NGS), Whole-genome transcriptional profile has become a great tool in transcriptome studies. Comparing with microarray methods, deep RNA sequencing (RNA-Seq) has been more widely used in different cell biology processes due to advantages of unbias, high-throughput, and sensitivity. The first transcriptome of *T. thermophila* was sequenced and released in 2012 (Wang et al., 2009; Xiong et al., 2012), which identified untranslated regions (UTR), novel transcripts and alternative splicing successfully and re-annotated *T. thermophila*’s genome more accurately. Performing RNA-Seq after knocking out genes could provide us a comprehensive understanding of the eliminated gene’s function in transcriptional level both in mammals and ciliates (Gao et al., 2013). This rapid and efficient method could be used as a powerful way to study further molecular mechanisms of interested genes.

While there has been a significant progress in understanding the behaviors of the *T. thermophila* proteins during meiosis and the possible interactions among them, this study was planned to uncover the functional role of cyc2p (*CYC2* protein) in a comprehensive and precise way and its possible involvement in meiotic recombination.

## Results

### Transcriptional expression profile of *CYC2*

**Figure 1:**
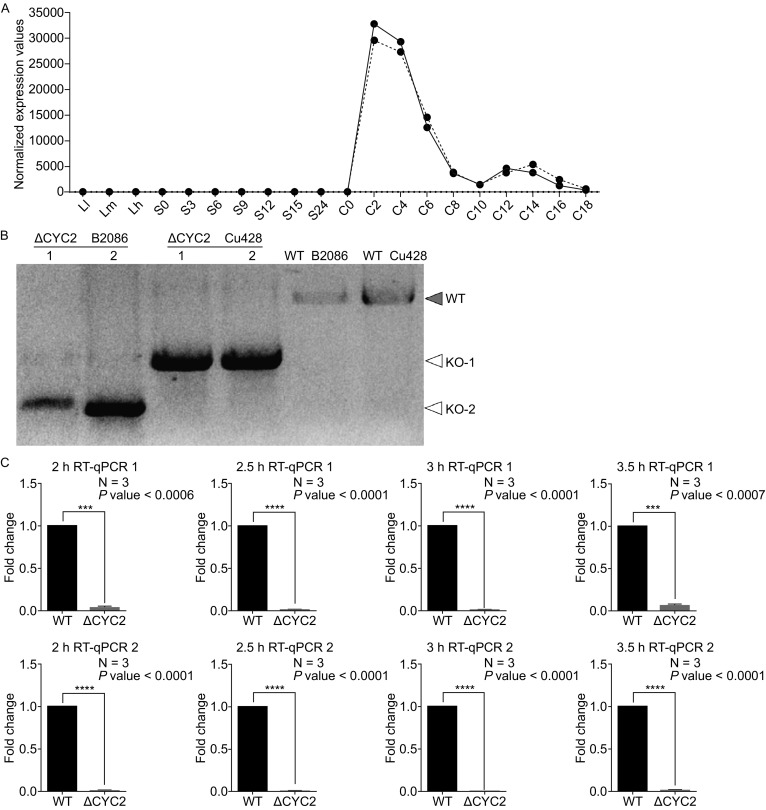
Transcriptional expression profile of *CYC2* (TTHERM_00079530) in *T. thermophila* and confirmation of *CYC2* knock-out strains. (A) The line chart was generated with microarray data of mRNA products of all time points (three GROWTH time points, seven STARVATION time points and ten conjugation time points) in life cycle of *T. thermophila*. The solid and dashed lines denoted the expression values normalized by two different methods. For vegetatively growing cells, L-l, L-m, and L-h respectively correspond to ~1 × 10^5^ cells/mL, ~3.5 × 10^5^ cells/mL, and ~1 × 10^6^ cells/mL. Samples collected at 0, 3, 6, 9, 12, 15, and 24 h after starvation began were respectively referred to as S-0, S-3, S-6, S-9, S-12, S-15, and S-24, and at 0, 2, 4, 6, 8, 10, 12, 14, 16, and 18 h after mixing equal volumes of B2086 and CU428 cells for conjugation initiation referred to as C-0, C-2, C-4, C-6, C-8, C-10, C-12, C-14, C-16, and C-18. It is manifested that *CYC2* expressed at transcriptional level exclusively during conjugation and abundantly during 2–4 h after conjugation initiation followed by additional expression around 12–14 h. (B) The *CYC2* knock-out strains (Δ*CYC2*) of two different mating types (B2086 and Cu428) were confirmed by whole cell extract PCR. The difference of PCR products lengths between strains of two mating types (marked as KO-1 and KO-2) was reasonable for a deleted region larger than target sequence, and it was often observed during the process of co-deletion (Hayashi and Mochizuki, 2015). (C) The fold changes of mRNA expression level of paired strains of Δ*CYC2* at 4 time points after conjugation initiation were examined by RT-qPCR using two different primer sets in CDS (‘qPCR mRNA check-1’ primers for RT-qPCR 1 and ‘qPCR mRNA check-2 exon1’ primers for RT-qPCR 2) (Fig. S1). N number of biological repeats

*CYC2* gene was selected from candidate genes which shared the similar variation tendency in mRNA expression profile with meiosis associated genes already known according to the analysis result from microarray and related RNA-Seq data. The mRNA expression profile is available on TGD website (http://ciliate.org/) where the microarray analysis results of genes at whole genome scale of *T. thermophila* are displayed (Miao et al., 2009). During logarithmic growth, when there are micronuclei mitosis and macronuclei amitosis, and starvation condition, *CYC2* gene doesn’t express at all. *CYC2* gene starts to express at the second hour after conjugation initiation and keeps a high expression level till the end of meiosis II (Fig. 1A).

### Deletion of *CYC2* caused the conjugation process arrest before crescent stage and initiation of meiosis I

The *CYC2* gene knock-out (KO) strains (Δ*CYC2*) of two mating types (CU428 and B2086 II) were previously constructed and well-tested (Hayashi and Mochizuki, 2015). We’ve also confirmed successful *CYC2* KO by whole cell extracts PCR at *CYC2* genomic locus (Fig. 1B) and the extreme low mRNA expression level of *CYC2* was also proved by RT-qPCR (Fig. 1C).

For phenotype assessment, Δ*CYC2* strains were starved for 24 h and mixed with equal amount of each mating type of strains to induce meiosis. Compared with wild type (control), we observed that almost none of the KO strain pairs had generated exconjugants which are the progeny cells after conjugation completely finished. Therefore, *CYC2* would be an essential gene in regulation cell cycle during meiosis process.

**Figure 2:**
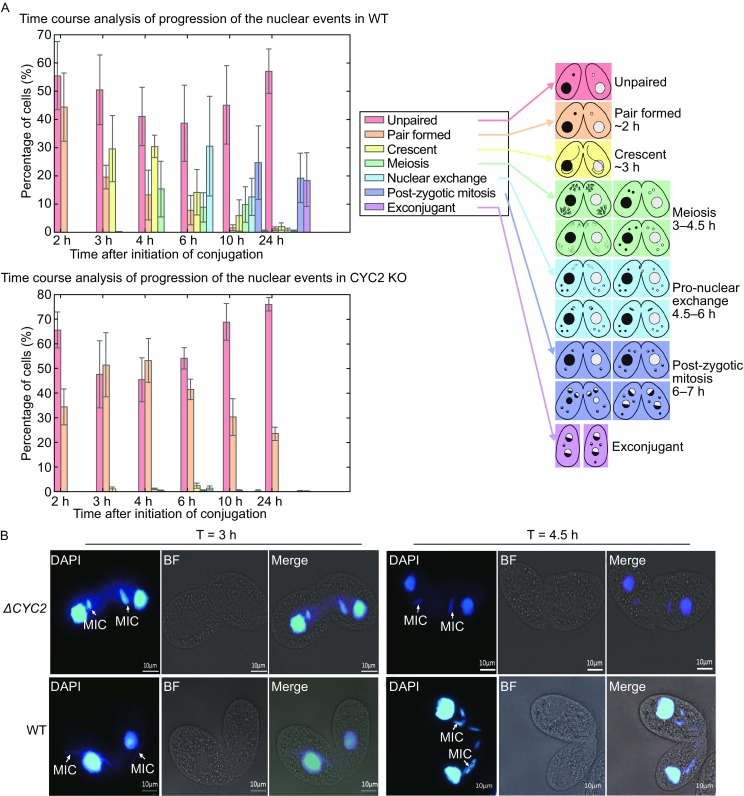
Phenotypic analysis of Δ*CYC2*. (A) The time course analysis of the progression of the nuclear events showed that the loss of *CYC2* resulted in an early conjugation arrest where the conjugants were not able to pass through the crescent stage successfully and lack of meiosis division. The conjugating process was artificially separated into seven parts according to the morphology characteristics (unpaired, pair formed, crescent, meiosis divisions, pro-nuclear exchange, post-zygotic mitosis, and exconjugant) and the related graphical representation were highlighted in different color at the right side. The 4 independent biological repeats of cell samples were collected respectively at 2, 3, 4, 6, 10, and 24 h after conjugation initiation. At least 100 pairs of cells were counted in each repeat. The percentage of each morphologic status was calculated and displayed in the histographs above. (B) The status of cell cycle arrest of Δ*CYC2* before crescent stage was captured during microscopic observation of cells stained with DAPI at 3 h when crescent shape formed in wild type conjugants and at 4.5 h when meiosis II was finished

The time line for progression of nuclear events in conjugation was assessed by DAPI staining (Fig. 2A). We found that at 1–2 h after the conjugation initiation, the KO strains could form normal pairs as wild type; and micronuclei could slightly migrate from macronuclei at around 2 h followed by stretching a little bit longer. The micronuclei were unable to form a crescent shape as wild type strains did in the beginning of meiosis. This process of stagnation persists till 4.5 h after conjugation initiation while the wild type pairs almost complete the meiosis process (Fig. 2B).

As the time goes on the KO strains pairs separated into single cells and the percentage of paired ones gradually decreased while the single unpaired cells increased. Eventually the majority of the KO pairs disconnected. These results showed us a meiosis disable phenotype after knocking out *CYC2* gene.

### RNA-Seq and data analysis of ΔCYC2

In order to test whether the *CYC2* gene affects the regulation of some meiosis associated genes at transcription level, we collected *CYC2* knocked out (ΔCYC2) and wild type cell samples at different four time points—2 h, 2.5 h, 3 h, and 3.5 h after conjugation initiation and perform a transcriptomic analysis by mRNA deep sequencing of RNA extraction products utilizing PolyA enrichment method. We obtained about 180 million paired-end reads, with a total length of more than 45 gigabases (GB). About 173 million (96%) of the paired-end reads could be mapped to the *T. thermophila* reference genome (Table 1). The expression level of each gene was represented as FPKM (Fragments per kilobases per million reads) with mapped reads according to previous reference genome annotation file.

**Table 1:** Statistics of RNA-seq reads

Sample name	Clean reads	Clean bases	Read length	Q20 (%)	Unique mapped reads
WT-2 h	44,363,694	5,545,461,750	125	96.60	42,626,886
WT-2.5 h	44,950,340	5,618,792,500	125	96.46	43,577,402
WT-3 h	45,183,552	5,647,944,000	125	96.06	43,300,024
WT-3.5 h	45,196,512	5,649,564,000	125	95.87	43,577,316
Δ*CYC2*-2 h	45,394,020	5,674,252,500	125	96.14	43,683,066
Δ*CYC2*-2.5 h	45,283,310	5,660,413,750	125	96.24	43,598,652
Δ*CYC2*-3 h	48,036,470	6,004,558,750	125	97.46	42,755,586
Δ*CYC2*-3.5 h	44,986,790	5,623,348,750	125	95.22	43,280,830
Total	363,394,688	45,424,336,000			346,399,762

**Figure 3:**
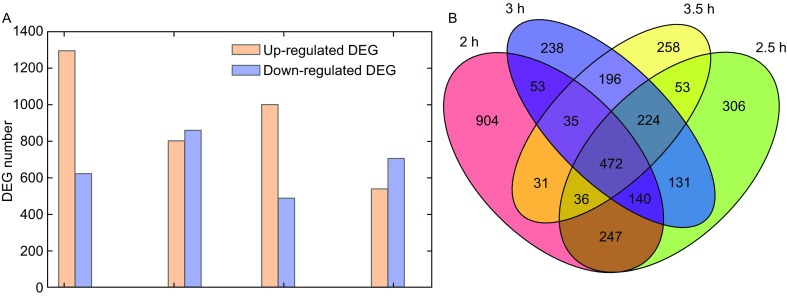
Overview of differentially expressed genes (DEGs). (A) DEG number of *CYC2* KO strains and wild type strains at four time stages (2, 2.5, 3, and 3.5 h after conjugation initiation). (B) Venn diagram identifying transcriptome DEG features between KO strains and wild type strains at four different time stages

The identified transcriptomes were compared between Δ*CYC2* strains and wild type strains at 2 h, 2.5 h, 3 h, 3.5 h four time stages, 1918, 1663, 1489, 1305 differentially expressed genes (DEGs) were identified (FDR < 0.05) respectively. We identified 1296 up-regulated DEGs, 622 down-regulated DEGs at 2 h stage, 803 up-regulated DEGs, 860 down-regulated DEGs at 2.5 h stage, 1001 up-regulated DEGs and 488 down-regulated DEGs at 3 h stage and 539 up-regulated DEGs, 706 down-regulated DEGs at 3.5 h stage (Fig. 3A). In addition to that, there was 479 genes differentially expressed at all four stages, and 895 genes differentially expressed at both 2 h and 2.5 h stages, 967 genes differentially expressed at both 2.5 h and 3 h stages, 927 genes differentially expressed at both 3 h and 3.5 h stages (Fig. 3B).

### Gene ontology enrichment analysis

**Figure 4:**
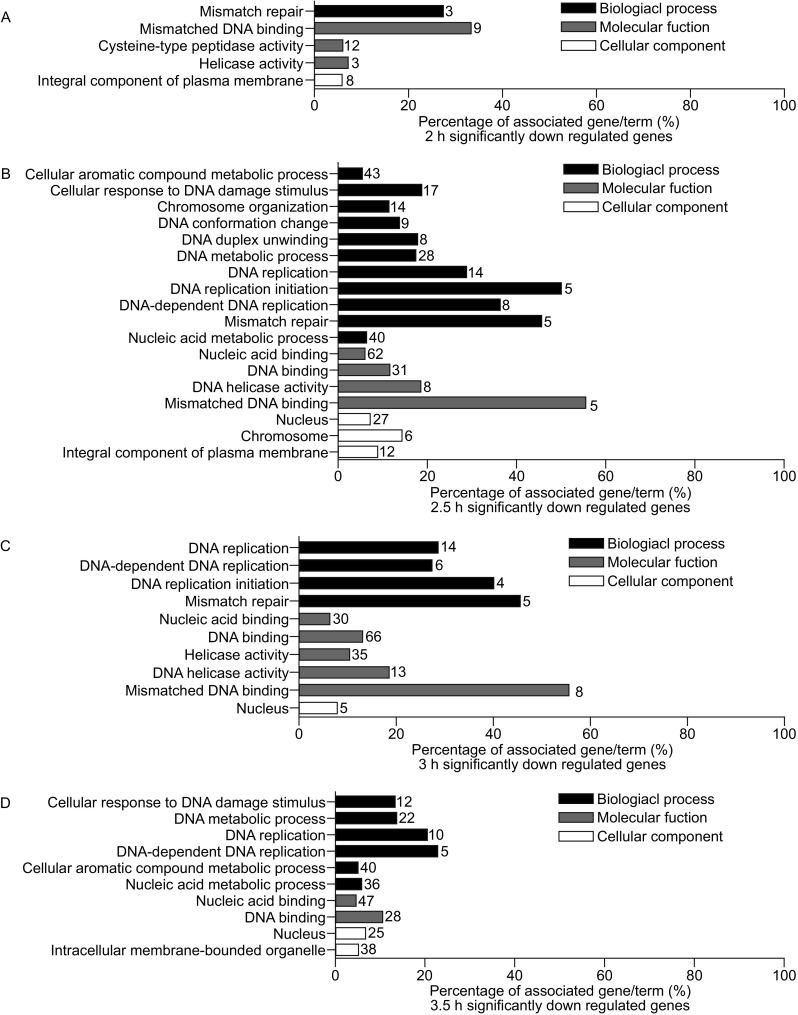
Gene ontology (GO) enrichment analysis of down regulated genes. The filtered significantly down regulated genes (Log2 (fold change) < −2 and *P* value < 0.05) of each time point were subjected to GO enrichment analysis separately in biological process, molecular biology, and cellular function. (A) Percentage of associated genes/term (%) at 2 h. (B) Percentage of associated genes/term (%) at 2.5 h. (C) Percentage of associated genes/term (%) at 3 h. (D) Percentage of associated genes/term (%) at 3.5 h. The length of bars from each histogram indicates the percentage of associated genes for each term. The number on top of each bar means the number of associated genes

The significantly up and down regulated genes at the four different time points were screened thoroughly according to the requirements that the binary logarithm of fold change should be no less than 2 for up regulated genes and no more than −2 for down regulated genes and *P* value no more than 0.05. The filtered data was used to perform the gene ontology (GO) enrichment analysis separately utilizing the ClueGO (Bindea et al., 2009) in three main groups: biological process, molecular function, and cellular component. The filtered DEGs up regulated at 2, 2.5, 3, and 3.5 h stages were enriched mostly in metabolic process related GO terms (Fig. S2), such as carbohydrate derivative metabolic process (GO: 1901135), carboxylic acid metabolic process (GO: 0019752). The filtered DEGs down-regulated at 2 h stage were enriched mainly in DNA MMR GO terms (Fig. 4A), like mismatch repair (GO: 0006298), mismatched DNA binding (GO: 0006298). At 2.5 h and 3 h stage, the filtered DEGs were enriched both in DNA MMR related GO terms and DNA replication related GO terms (Fig. 4B and 4C), such as DNA replication (GO: 0006298), cellular response to DNA damage (GO: 0034984). Also the filtered DEGs at 3.5 h stage were enriched in DNA replication related GO terms like DNA replication (GO: 0006298), DNA-dependent DNA replication (GO: 0006262) (Fig. 4D). The full GO enrichment analysis results were supplemented in Table S2–S5.

Together, from the GO analysis results of the up regulated DEGs (Fig. S2) there were little strong enrichment at any time point of the four. The results at each of the four time points showed that biological process and molecular function almost centered on various metabolic process of organic compounds and that the protein products of these genes were dispersed into different cellular components without well-detected focus. In sharp contrast to the situation in the up regulated DEGs, the significantly down-regulated part manifested a strong enrichment from the angle of biological process and molecular function in DNA MMR and DNA replication, while it is mainly enriched in nucleus in aspect of cellular component, which may indicate that knocking out of *CYC2* may result in DNA MMR and DNA replication related genes’ dysfunction, further causing Δ*CYC2* strains’ meiosis disability.

### The affected meiosis associated genes in ΔCYC2 strain

The 60 best-conserved *Tetrahymena* meiotic proteins used as a criteria for a possible meiosis-specific function was previously manifested (Mochizuki et al., 2008). We observed that 20 of these 60 proteins (Table 2) were significantly affected upon KO of cyc2p indicating that around one third of the known meiosis-associated genes were transcriptionally associated with *CYC2*. Among these genes, *SPO11* (TTHERM_00627090), which plays a significant role in meiotic homolog recombination as it catalyzes DSB formation (Mochizuki et al., 2008), was drastically down regulated at the fold change of −16. *IME4* (TTHERM_00962190), a homolog of yeast gene that is required for entry into meiosis (Shah and Clancy, 1992) was significantly up regulated at the 2.5 h stage. *CYC24* (TTHERM_00842480), *CYC6* (TTHERM_00194440), and *CYC18* (TTHERM_00827080) which might account for regulation of cell cycle progression in meiosis (Mochizuki et al., 2008) were up regulated at 3.5 h stage.

**Table 2:** Significantly regulated meiosis associated genes after *CYC2* KO

Gene ID	Meiotic functions	Family name	Standard name of *T. thermophila*	Discription	2 h	2.5 h	3 h	3.5 h
TTHERM_00962190	Meiotic induction	Ime4	IME4 (Homolog of budding yeast IME4 (Inducer of meiosis))	In yeast, required for entry into meiosis\nMT-A70 family protein	0.463063	1.95484	1.51113	0.963928
TTHERM_00842480	Regulation of cell cycle progression	Cyclin	CYC24 (CYClin)	Cyclin, N-terminal domain containing protein	0.81999	1.72414	2.11614	2.01071
TTHERM_00194440	Regulation of cell cycle progression	Cyclin	CYC6 (CYClin)	Cyclin, N-terminal domain containing protein	3.27593	2.20212	2.18551	2.44223
TTHERM_00827080	Regulation of cell cycle progression	Cyclin	CYC18 (CYClin)	Cyclin, N-terminal domain containing protein	2.70469	2.74254	2.16665	2.41128
TTHERM_00627090	DSB formation	Spo11/Rec12	SPO11 (Ortholog of budding yeast SPO11)	Type IIB DNA topoisomerase family protein required for meiotic DNA DSBs. Required for the elongation of meiotic nuclei and full chromosome pairing in *Tetrahymena*	−4.28045	−4.81505	−4.71778	−4.31692
TTHERM_00459230	Strand exchange	Dmc1	DMC1 (DMC1 homolog)	meiosis-specific RecA homolog	−4.15141	−4.85911	−4.68173	−4.49637
TTHERM_00721450	Recombinational repair	Mre11/Rad32	MRE11 (Homolog of budding yeast MRE11 and of fission yeast RAD32)	Ser/Thr protein phosphatase family protein. Mre11p is required for the repair of meiotic double-strand breaks and full chromosome pairing	−0.555912	0.128007	1.20542	1.88526
TTHERM_01179960	DSB repair	Exo1	EXO1 (Homolog of budding yeast, fission yeast and mouse EXO1)	XPG I-region family protein involved in DSB repair	−0.224175	−1.9432	−2.19387	−2.02446
TTHERM_00011650	DSB repair	Rad10	RAD10 (Homolog of budding yeast RAD10)	In yeast, single-stranded DNA endonuclease (with Rad1p), cleaves single-stranded DNA during nucleotide excision repair and double-strand break repair	1.56953	−0.692742	−2.38111	−1.50885
TTHERM_01109940	Mismatch repair	Pms1	PMS2 (DNA mismatch repair also called pms1)	DNA mismatch repair protein, C-terminal domain containing protein; homolog to human PMS2	−2.05342	−2.63375	−2.38925	−1.71021
TTHERM_00194810	Mismatch repair	Msh6	MSH6 (homolog to human protein MSH6)	MutS domain III family protein	−1.80791	−2.75677	−2.25144	−1.89783
TTHERM_00426230	Mismatch repair	Msh3	MSH3 (homolog to human protein MSH3)	MutS domain III family protein	−0.461977	−2.16236	−2.1011	−1.67332
TTHERM_01030000	Regulates crossing over	Sgs1/Rqh1	SGS1 (Homolog of budding yeast Sgs1 and fission yeast rqh1)	ATP-dependent DNA helicase, RecQ family protein involved in DNA joint molecule resolution dHJ dissolution and crossing over	−2.14875	−2.88759	−2.53104	−1.75497
TTHERM_00794620	Strand exchange	Hop2/Meu13	HOP2 (Meiosis-specific homolog of budding yeast Homologous Pairing 2)	HOP2 has a role in chiasmata and meiotic bivalent formation. There exists a ubiquitously expressed paralog, TTHERM_01190440 (HOPP2), which is essential for vegetative growth	−3.07189	−3.80692	−2.88744	−2.01004
TTHERM_00300660	Strand exchange	Mnd1/Mcp8	MND1 (Homolog of budding yeast MND1 (meiotic non disjunction) and fission yeast MCP7)	In budding yeast, the Mnd1 protein forms a complex with Hop2 to promote homologous chromosome pairing and meiotic DSB repair. Mnd1 requires Hop2 to localize to chromosomes	−0.581187	−2.04827	−1.82826	−1.75667
TTHERM_00441940	APC regulator	Fzr1/CDH2	FZY9 (Fizzy)	fizzy/CDC20/CDH1 family protein; homolog of *S. cerevisiae* CDH1, a cell cycle regulated activator of the anaphase-promoting complex/cyclosome (APC/C), which directs ubiquitination of various targets	−0.0326336	0.687104	1.94776	2.2213
TTHERM_00297160	–	Cut1	ESP1 (Extra Spindle Pole bodies)	Separase protein, required for mitotic and meiotic chromosome segregation	−1.64457	−3.70644	−3.01699	−2.45854
TTHERM_00158460	–	Mei2	RRM68 (RNA recognition motif-containing protein 68)	RNA recognition motif 2 family protein	−1.76957	−2.30628	−1.55013	−1.17294
TTHERM_00684590	–	Aurora kinases	None	Protein kinase domain containing protein. Sequence similarity to the Aurora protein kinase family	−2.31013	−3.86966	−3.97498	−3.74671
TTHERM_00991560	–	–	None	Protein phosphatase 2A regulatory B subunit (B56 family)	3.49729	5.07489	2.99617	2.78042

Numbers in last four columns (2 h, 2.5 h, 3 h, 3.5 h) mean log2 fold change of KO/WT RPKM of related genes, bolded numbers are significant statistically

The following genes were also down regulated in Δ*CYC2*: *RAD51*, TTHERM_00142330; *DMC1*, TTHERM_00459230; *SGS1*, TTHERM_01030000; *HOP2*, TTHERM_00794620; *MND1*, TTHERM_00300660) which responsible for strand exchange during crossing over and (*RFA1*, TTHERM_00106890; *MRE11*, TTHERM_00721450; *EXO1*, TTHERM_01179960; *PMS2*, TTHERM_01109940; *TMLH1*, TTHERM_00127000; *MSH6*, TTHERM_00194810; *MSH3*, TTHERM_00426230. They were reported to be responsible for DNA damage repair especially mismatch repair (Mochizuki et al., 2008). The expression of *ESP1* (TTHERM_00297160) and a putative Aurora protein kinase (TTHERM_00684590), which may involve in spindle assembly and chromosome separation at the anaphase of meiosis (Mochizuki et al., 2008), were also down regulated. The large percentage of significantly regulated genes in the listed meiotic specific genes added weight to our view point that *CYC2* was probably involved in the meiosis associated regulation network.

### Disable meiotic DSB formation in Δ*CYC2* strain

**Figure 5:**
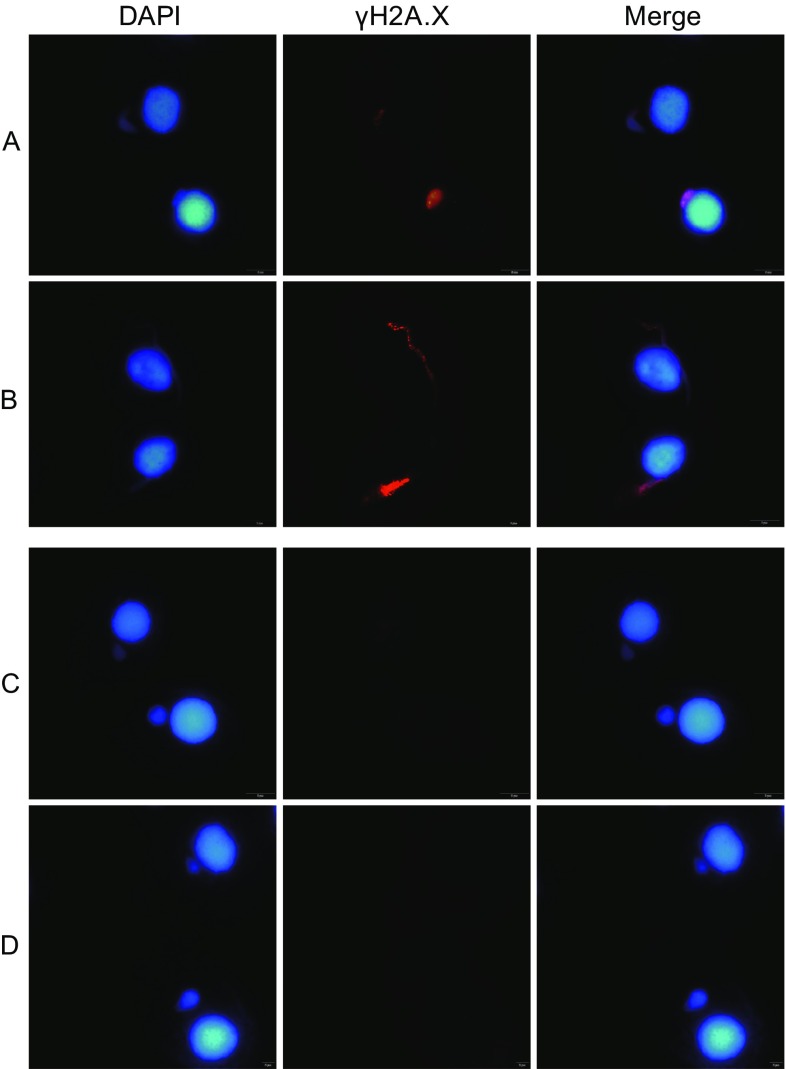
Immunostaining of wild type and *CYC2* KO strains. (A) Gamma-H2AX signals occupied the whole wild type micronuclei before crescent stage. (B) Gamma-H2AX signals occupied the whole wild type meiotic micronuclei during crescent stage. (C and D) There were no gamma-H2AX signals in *CYC2* KO micronuclei, which could not elongate fully during conjugation

The presence of the phosphorylated histone H2A.X variant (gamma-H2A.X) is a marker for the location of DSBs appears on the meiotic chromosomes of *T. thermophila* (Song et al., 2007). We test the formation of meiotic DSBs by performing immunofluorescence of gamma-H2AX. In wild type, meiotic DSBs begin before crescent stage when micronuclei haven not elongated (Fig. 5A) and also were seen at crescent stage (Fig. 5B). In contrast, we did not detect any DSB signal in micronuclei of *CYC2* KO strains, which also could not elongate into crescent (Fig. 5C and 5D). These results suggest that meiotic DSB formation was totally disrupted after knocking out of the *CYC2* gene.

### Validation of the RNA-Seq results

**Figure 6:**
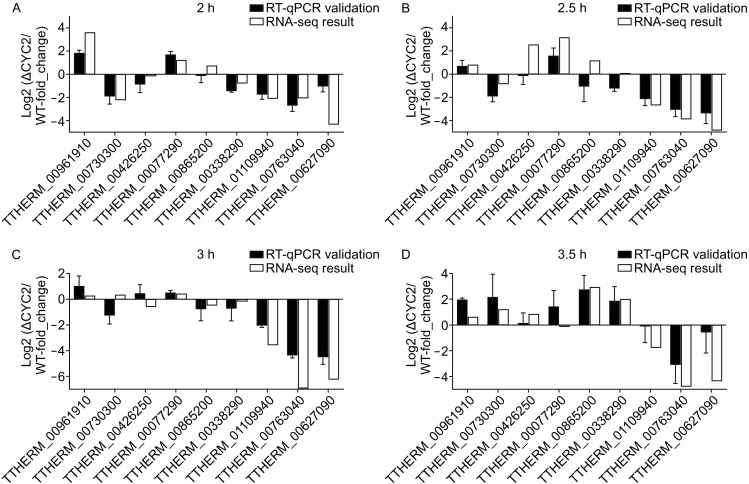
RT-qPCR verification of the RNA-Seq results. The 8 randomly selected genes (TTHERM_00961910; TTHERM_00730300; TTHERM_00426250; TTHERM_00077290; TTHERM_00865200; TTHERM_00338290; TTHERM_01109940; TTHERM_00763040) plus *CYC2* TTHERM_00079530) and *SPO11* (TTHERM_00627090) were performed with RT-qPCR. The primers were designed to across an intron to eliminate potential confluence from residue genomic DNA contamination. cDNA templates were from of 3 independent biological repeats of cell samples respectively at 2 h (A), 2.5 h (B), 3 h (C), and 3.5 h (D) after conjugation initiation. The tendencies of mRNA expression changes were in accordance with the RNA-Seq analysis results, which well-validated of the sequencing data

From the significantly regulated genes of four time points after conjugation initiation we randomly selected 9 genes together with *SPO11* gene to check the validity and credibility of RNA-Seq results by comparing the mRNA expression profile of 3 independent biological repeats of conjugants of wild type and *CYC2* KO strains, using reverse transcription quantitive PCR (RT-qPCR) with *RPL11* as the internal control (Fig. 6). The list of these 9 genes with some annotation details and the binary logarithm of fold changes of being regulated can be viewed in supplement archive. Despite some slight differences, the general expression profiles were quite consistent between the RNA-Seq and RT-qPCR data, convincingly validating the reliability of the transcriptomic changes after knocking out *CYC2.*

## Discussion

*T. thermophila* is a great model organism for studying meiosis process, since its meiosis property make synchronization easy and high-efficient. Previous work mainly conducted traditional biochemistry and cell biology methods to study meiosis process of this ciliate (Howard-Till et al., 2011). In our research, we highlight on meiosis process by combining phenotype analysis and transcriptome-wide deep sequencing with traditional biochemistry methods. RNA-Seq analysis was performed to determine the effects of *CYC2* KO on genome-wide genes’ expression levels during meiosis. Plenty of DEGs between *CYC2* KO and wild type strains were functionally enriched in DNA mismatch repair and DNA replication field, revealing that cyc2p, a cell cycle related protein, would be essential to meiosis by participating in DSBs formations, DNA mismatch repair and replication.

On this experimental design, Δ*CYC2* displayed conspicuously defect of meiotic micronuclei elongation and meiotic DSBs formation in compatible with previously published phenotype of *SPO11* knockout strains (Δ*SPO11*) during the prophase of meiosis I (Mochizuki et al., 2008; Zhang et al., unpublished data). Ample evidence indicates that DSBs are initiators of meiotic recombination. We reasoned that the absence of DSBs formation in Δ*CYC2* strain was due to the drastic reduction in *SPO11* expression, which is responsible for formation of meiotic DSBs as catalyzing the DNA cleavage via its Type II B topoisomerase-like transesterase activity with the help of a series of other related proteins (Bergerat et al., 1997; Cao et al., 1990; Keeney et al., 1997b; Lam and Keeney, 2015; Sun et al., 1989; Szostak et al., 1983).

Some previous research reported that spo11p in mice and budding yeast might function independently of DSBs induction to assist homolog alignment (Boateng et al., 2013; Loidl, 2013). Thus, spo11p has very crucial roles in inducing DNA self-generated DSBs, which is indispensable for homolog chromatin crossover formation at synaptonemal complex state, and possibly also in promoting homologs to approach each other to be aligned faithfully. That may be reasonable to explain the act in *T. thermophila* of the germline nuclear elongation since the homolog recognition is easy to achieve when chromosome become a long thin bouquet in crescent stage.

However, although *SPO11* is the catalytic center of the meiotic recombination initiation mechanism, and is also highly expressed at 2–4 h after conjugation initiation and involved in DSBs formations, *CYC2* gene’s expression has not been affected by knocking out *SPO11* according to TGFD (*Tetrahymena* Functional Genomics Database) meiosis RNA-Seq data (Fig. S3) and (Zhang et al., unpublished data). This could indicate that *CYC2* may play a key role in regulating *Spo11* and further influencing the whole downstream process of meiosis.

Additionally, HR is associated very closely with DNA repair including mismatch repair (Spies and Fishel, 2015). Previous study has demonstrated that the recombinases Rad51 and Dmc1 play important roles in DNA break repair and recombination partner choice in the meiosis of *Tetrahymena* (Howard-Till et al., 2011). Also during the streamlined meiosis of *Tetrahymena*, Msh family protein function in synaptonemal complex independent chiasma formation (Shodhan et al., 2014). A very interesting phenomenon we observed is that several key meiotic recombination proteins associated with DNA repair (*MRE 11*, *RAD51*, *DMC1*, *Msh3*, and *Msh6*), which were all down regulated in *CYC2* KO (Table 2), were not affected after ablating *SPO11* according to TGFD meiosis RNA-Seq data (Zhang et al., unpublished data). Also the recombination defect in *SPO11* mutants can be partially rescued by production of DSBs from an exogenous source such as ionizing radiation (Loidl and Mochizuki, 2009). These data may confirm the involvement of *SPO11* in DSBs formation without effects on meiotic recombination machinery proteins. Meanwhile these could indicate that DNA repair (including MMR) genes down regulated in Δ*CYC2* may be also directly regulated by *CYC2* rather than caused through *SPO11* down-regulated.

In the present study we concluded that the meiotic disabled phenotypes and the absence of DSBs in Δ*CYC2* strains due to the down regulation of *SPO11* but the exact mechanism how *CYC2* actively control *SPO11* at the right time and place remains unknown. Also, these data will encourage us to unravel the pathway how *CYC2* affect meiotic recombination process during meiosis I prophase. Another challenge is the need to fully catalog *CYC2* in *T. thermophila* and consider the following questions concerning the initiation of meiotic recombination, for example, how does *CYC2* actively regulate *SPO11* to promote DSBs formation? How does *CYC2* regulate the recombination machinery genes or proteins? Does *CYC2* act as a meiosis specific transcription factor?

## Materials and methods

### *T. thermophila* strains and culture conditions

WT strains CU428 and B2086 II were provided by P. J. Bruns (Cornell University, Ithaca, NY, USA, now available through the National *Tetrahymena* Stock Center, http://tetrahymena.vet.cornell.edu/index.html). Cells were grown in super proteose peptone (1× SPP) medium (1% proteose peptone, 0.2% glucose, 0.1% yeast extract, and 0.003% EDTA ferric sodium salt) at 30°C with vigorous shaking (rotation speed usually at 180–220 rpm), unless otherwise specifically requested. For starvation, log phase cells were washed and resuspended in 10 mmol/L Tris (pH 7.4) at 30°C without shaking for 24 h unless specified otherwise. Cells were counted using a hemocytometer to control the concentration of the strains (Gorovsky et al., 1975).

### Phenotypic analysis

Two mating types (CU428 VII and B2086 II) of *T. thermophila* were fully starved by washing and resuspending in 10 mmol/L Tris (pH 7.4) in flasks without shaking for 20–24 h at 30°C, followed by mixing almost equal quantities of each to artificially start the conjugation process. Several time points interested were chose and the corresponding cell samples were collected, fixed with formaldehyde and stained with DAPI. Each sample was observed by Olympus fluorescence microscope to be classified to different stages by the developmental characteristics of both the Micronucleus and Macronucleus during conjugation and counted many enough cells to conclude the statistical number proportion.

### Transcriptome sequencing

Cells for each of the samples with the amount of approximately 1 × 10^7^ (cells) for each samples were washed once with 1× PBS (centrifuge at 450 ×*g* for 2 min) and spun down to pellet followed by immediate freezing in liquid nitrogen. After the total RNA extraction and DNase I treatment, magnetic beads with Oligo (dT) are used to isolate mRNA. Mixed with the fragmentation buffer, the mRNA is fragmented into short fragments. Then cDNA is synthesized using the mRNA fragments as templates. Short 6/8 fragments are purified and resolved with EB buffer for end reparation and single nucleotide A (adenine) addition. After that, the short fragments are connected with adapters. The suitable fragments are selected for the PCR amplification as templates. During the QC steps, Agilent 2100 Bioanaylzer and ABI Step One Plus Real-Time PCR System are used in quantification and qualification of the sample library. At last, the libraries are sequenced using Illumina HiSeq 2000.

### Data processing, identified DEGs and GO enrichment analysis

Raw sequencing reads were trimmed by removing Illumina adapter sequences and low quality bases. The STAR program with default parameters was used to map the sequenced reads to the reference genome and to find those that spanned exon–exon junctions. Transcripts were assembled using the Cufflinks software and gene expressions were based on fragments per kilobase of exon model per million mapped reads (FPKM) values. FPKM of different samples were compared by using Cuffdiff to identify DEGs. The up-regulated and down-regulated DEGs were analyzed for gene ontology (GO) through ClueGO (v 2.1.7) software. The RNA-Seq data used in this study have been deposited in NCBI Gene Expression Ommbius (GSE79286).

### Indirect immunofluorescence microscopy

1.5 mL of mating cells with 2 × 10^5^ cells/mL density in 10 mmol/L Tris, pH 7.5 were mixed with 5 μL of partial Schaudin’s fixative (two parts saturated HgCl_2_ to one part 100% ethanol) and incubated for 5 min at room temperature (RT). Cells were gently pelleted (130 ×*g* for 30 s) and washed once with 3 mL of RT methanol and resuspended in 1 mL of RT methanol. 50 μL of cells suspension was spread onto a coverslip and air dried for 30 min. Cells were stained with anti-γH2A.X (1:100) (Anti-phospho-H2A.X (Ser139) Mouse Ab (Millipore 05-636-I) Antibody) followed by incubation with AlexaFluor 568 goat anti-mouse immunoglobulin G (IgG; 1:400) (Invitrogen). Nuclei were stained with the DNA-specific dye 4′,6′-diamidino-2-phenylindole (DAPI; Roche) at 10 ng/mL for 10 min. Images were obtained with an Olympus IX73 fluorescence microscope.

### Isolation of total cellular RNA

The total cellular RNA of all samples in this study were extracted using Eastep^TM^ Universal RNA Extraction Kit (Promega, Cat.No.LS1030) followed by reverse transcription using GoScript™ Reverse Transcription System Kit (Promega, Cat.No.A5000) and the step by step procedures are based on the given product manuals all available online.

### RT-qPCR

The cDNA products from RNA extraction and reverse transcription underwent real time quantitive PCR to quantify the mRNA expression level of selected genes using AceQ^®^ qPCR SYBR^®^ Green Master Mix (Vazyme, Cat.No.Q111-03) on the equipment CFX Connect Bio-Rad. The procedures in details and the PCR thermocycle protocol are based on the given product manuals all available online.

## Electronic supplementary material

Below is the link to the electronic supplementary material.

Supplementary material 1 (XLSX 9 kb)

Supplementary material 2 (XLSX 14 kb)

Supplementary material 3 (XLSX 16 kb)

Supplementary material 4 (XLSX 19 kb)

Supplementary material 5 (XLSX 17 kb)

Supplementary material 6 (XLSX 10 kb)

Supplementary material 7 (PDF 598 kb)
