# Correction to: The key role of *CYC2* during meiosis in *Tetrahymena Thermophila*

**DOI:** 10.1007/s13238-018-0562-3

**Published:** 2018-06-27

**Authors:** Qianlan Xu, Ruoyu Wang, A. R. Ghanam, Guanxiong Yan, Wei Miao, Xiaoyuan Song

**Affiliations:** 1 Hefei National Laboratory for Physical Sciences at the Microscale, University of Science and Technology of China, Hefei, 230026 China; 2 CAS Center for Excellence in Molecular Cell Science, Shanghai, 200031 China; 3 School of Life Sciences, University of Science and Technology of China, Hefei, 230071 China; 4 Key Laboratory of Aquatic Biodiversity and Conservation, Institute of Hydrobiology, Chinese Academy of Sciences, Wuhan, 430072 China; 5 University of Chinese Academy of Sciences, Beijing, 100049 China; 6 Anatomy and Embryology Department, Suez Canal University, Ismailia, 41522 Egypt

## Correction to: Protein Cell 2016, 7(4):236–249 10.1007/s13238-016-0254-9

In the original publication, the funding information was incorrectly published. The correct funding information is provided in this correction.

This work is supported by grants from the Projects of International Cooperation and Exchanges Ministry of Science and Technology of China (2013DFG32390) and the National Natural Science Foundation of China (31472059) to X.S. X.S is a recipient of the Young Thousand Talents program (KJ2070000026).

