# Supplementary material for: The key role of CYC2 during meiosis in Tetrahymena thermophila
Source: Protein Cell. 2016 Mar 23;7(4):236–49. doi: 10.1007/s13238-016-0254-9 (PMC4818844; doi:10.1007/s13238-016-0254-9)
Supplement: Supplementary file 7 — Supplementary material 7 (PDF 598 kb) [file 13238_2016_254_MOESM7_ESM.pdf]

(l) Supplementary materials

Supplementary tables (Table S1-S6) were in the format of MS Excel forms and uploaded separately.

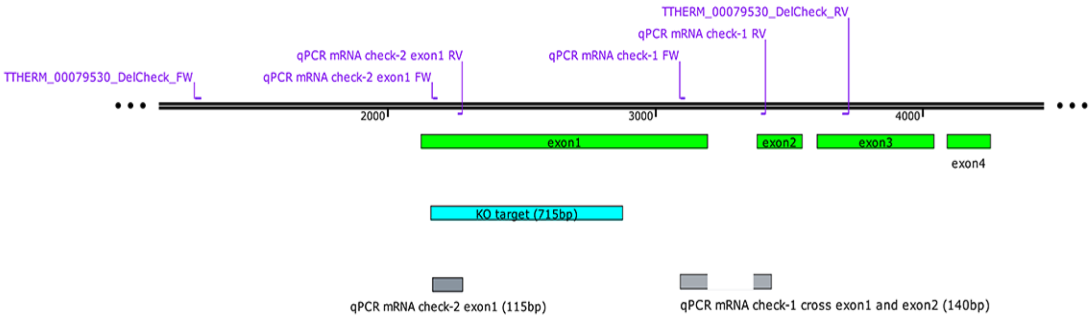

Supplementary Figure 1

Primer sets in UTR and CDS of TTHERM_00079530 (*CYC2*): KO target 715bp: Knock-out target sequence was 715bp long in exon1 in CDS of TTHERM_00079530. TTHERM_00079530_DelCheck_FW and TTHERM_00079530_DelCheck_FW were primers for whole cell extract PCR **(Figure 1B)**. qPCR mRNA check-1 FW and qPCR mRNA check-1 RV were primers for RT-qPCR product across exon1 and exon2 of *CYC2* CDS and qPCR mRNA check-2 exon1 FW and qPCR mRNA check-2 exon1 RV were primers for RT-qPCR product in exon1.

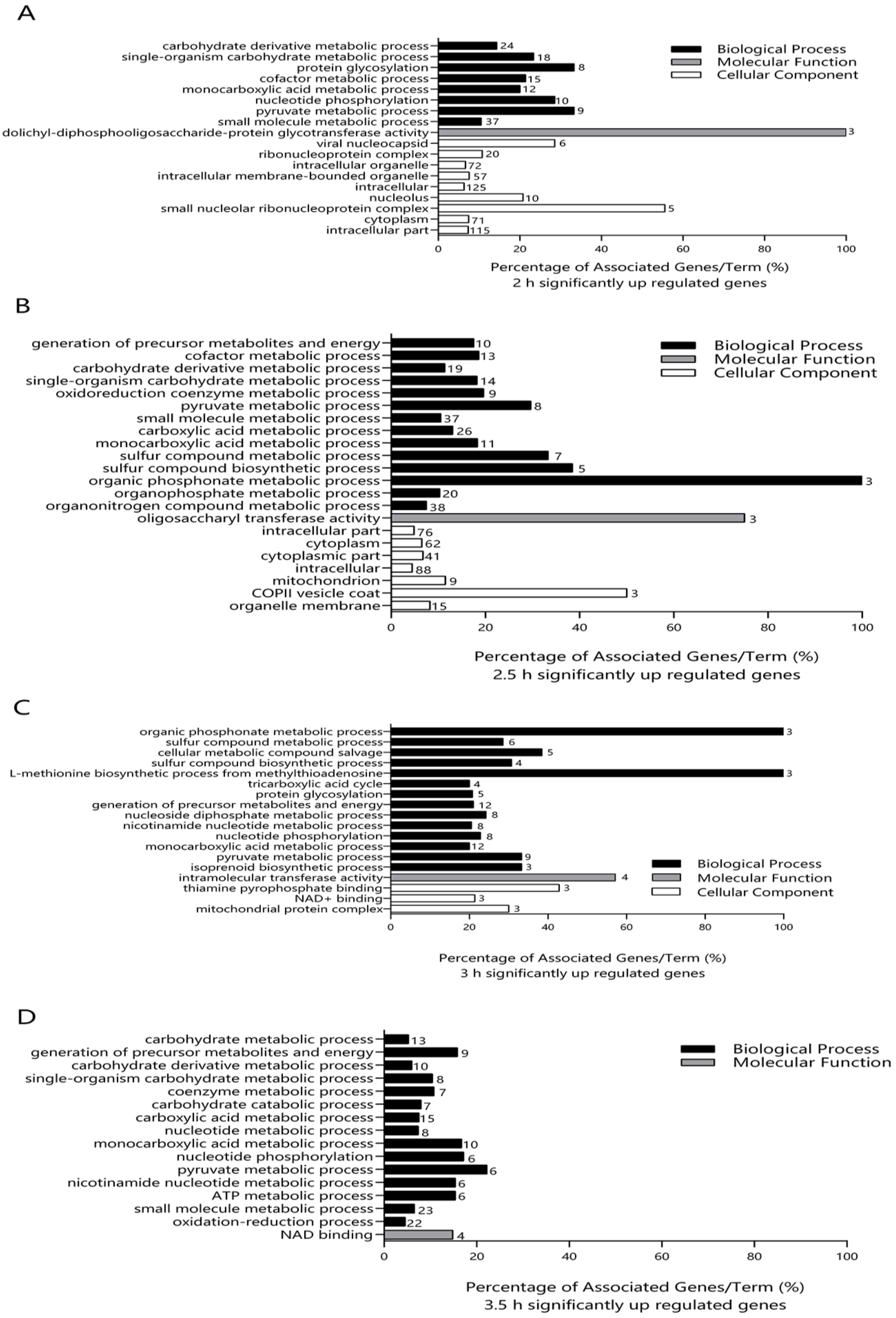

A
carbohydrate derivative metabolic process
single-organism carbohydrate metabolic process
protein glycosylation
cofactor metabolic process
monocarboxylic acid metabolic process
nucleotide phosphorylation
pyruvate metabolic process
small molecule metabolic process
dolichyl-diphosphooligosaccharide-protein glycotransferase activity
viral nucleocapsid
ribonucleoprotein complex
intracellular organelle
intracellular membrane-bounded organelle
intracellular
nucleolus
small nucleolar ribonucleoprotein complex
cytoplasm
intracellular part
24
18
8
15
12
10
9
37
3
6
20
72
57
125
10
5
71
115
Biological Process
Molecular Function
Cellular Component
0
20
40
60
80
100
Percentage of Associated Genes/Term (%)
2 h significantly up regulated genes
B
generation of precursor metabolites and energy
cofactor metabolic process
carbohydrate derivative metabolic process
single-organism carbohydrate metabolic process
oxidoreduction coenzyme metabolic process
pyruvate metabolic process
small molecule metabolic process
carboxylic acid metabolic process
monocarboxylic acid metabolic process
sulfur compound metabolic process
sulfur compound biosynthetic process
organic phosphonate metabolic process
organophosphate metabolic process
organonitrogen compound metabolic process
oligosaccharyl transferase activity
intracellular part
cytoplasm
cytoplasmic part
intracellular
mitochondrion
COPII vesicle coat
organelle membrane
10
13
19
14
9
8
37
26
11
7
5
3
20
38
3
76
62
41
88
9
3
15
Biological Process
Molecular Function
Cellular Component
0
20
40
60
80
100
Percentage of Associated Genes/Term (%)
2.5 h significantly up regulated genes
C
organic phosphonate metabolic process
sulfur compound metabolic process
cellular metabolic compound salvage
sulfur compound biosynthetic process
L-methionine biosynthetic process from methylthioadenosine
tricarboxylic acid cycle
protein glycosylation
generation of precursor metabolites and energy
nucleoside diphosphate metabolic process
nicotinamide nucleotide metabolic process
nucleotide phosphorylation
monocarboxylic acid metabolic process
pyruvate metabolic process
isoprenoid biosynthetic process
intramolecular transferase activity
thiamine pyrophosphate binding
NAD+ binding
mitochondrial protein complex
3
6
5
4
3
4
5
12
8
8
8
12
9
3
4
3
3
3
Biological Process
Molecular Function
Cellular Component
0
20
40
60
80
100
Percentage of Associated Genes/Term (%)
3 h significantly up regulated genes
D
carbohydrate metabolic process
generation of precursor metabolites and energy
carbohydrate derivative metabolic process
single-organism carbohydrate metabolic process
coenzyme metabolic process
carbohydrate catabolic process
carboxylic acid metabolic process
nucleotide metabolic process
monocarboxylic acid metabolic process
nucleotide phosphorylation
pyruvate metabolic process
nicotinamide nucleotide metabolic process
ATP metabolic process
small molecule metabolic process
oxidation-reduction process
NAD binding
13
9
10
8
7
7
15
8
10
6
6
6
6
23
22
4
Biological Process
Molecular Function
0
20
40
60
80
100
Percentage of Associated Genes/Term (%)
3.5 h significantly up regulated genes

Supplementary Figure 2

Gene Ontology (GO) enrichment analysis of up regulated genes: The filtered significantly up regulated genes (Log2 (fold change) >2 and P value <0.05) of each time point were subjected to GO enrichment analysis separately in biological process, molecular biology and cellular function. (A) Percentage of associated genes/term (%) at 2 h. (B) Percentage of associated genes/term (%) at 2.5 h. (C) Percentage of associated genes/term (%) at 3 h. (D) Percentage of associated genes/term (%) at 3.5 h. The length of bars from the each histogram indicates the percentage of associated genes for each term. The number on top of each bar means the number of associated genes.

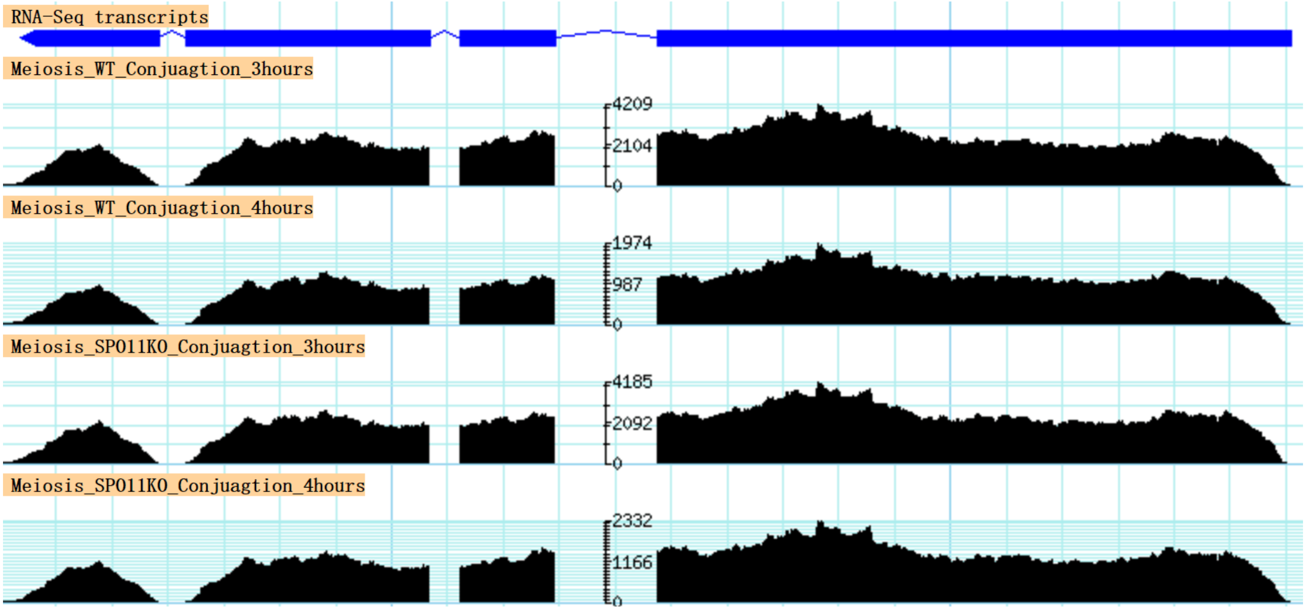

Supplementary Figure 3

RNA-Seq Data Tracks From TFGD (Tetrahymena Functional Genomics Database). The First track shows the CYC2 gene transcripts locus in genome browser. Other tracks show the transcription profile of CYC2 gene in WT 3 h, WT 4h, SPO11 KO 3 h, SPO11 KO 4 h.
